# Dislocation of the Shoulder, of Eight Weeks Standing, Reduced

**Published:** 1844-06

**Authors:** Daniel Brainard


*Dislocation of the Shoulder, of eight weeks standing, reduced.* By DANIEL BRAINARD, M. D.

Elisha Pratt, of Deer Grove, Ill., aged 43 years, of relaxed habit, came to me, May 5th, 1843, with an unreduced dislocation of the left shoulder, of 56 days standing. The head of the humerus was situated before and on the inner side of the glenoid cavity, resting against the coracoid process of the scapula and was distinctly felt with the hand in the axilla. The natural roundness of the shoulder was lost; there was a depression beneath the acromion process; the elbow was carried from the side, and the movements of the member limited, in a word the existence of the dislocation was easily recognized. The patient desiring its reduction, it was attempted and performed in the following manner: He was placed upon his back on a low table, extension made with the pulleys, from a roller passed about the arm above the elbow, counter-extension being made by a long band passed around the thorax under the axilla and secured by a narrower piece of linen tied over the scapula. The pulleys, and the counter-extending band, were fixed to staples in the walls. The force was applied in such a direction as to draw the arm horizontally, and at a right angle with the trunk. For the purpose of relaxation of the system tart. ant. et potass was administered to nausea, and when the extension was comnenced, a vein in the arm was opened and blood drawn to approaching syncope. As soon as extension had been commenced, the arm was rotated freely and the head of the humerus pressed in different directions, in order to break up its newly formed attachments. These gradually gave way, the sound from their separation being sufficiently loud to be perceived by all the assistants. The force required to change the position of the bone was very considerable; at length, however, this was effected, sufficiently so at the end of 55 minutes to allow of its replacement in the socket, which was effected by suddenly detaching the pulleys, and bringing the elbow to the body while the knee was in the axilla. A pad was then placed in the axilla and the elbow retained at the side by a roller.

Some ecchymosis and inflammation were present the next day, for which evaporating lotions were applied and a saline purge administered. At the end of a week he returned home, the swelling having subsided, and the bone having no tendency to displacement, the elbow however was still kept in a sling, and the patient cautioned against extended movements of the limb.

The question of the propriety of efforts at reduction of ancient dislocations, especially those of the superior extremity of the humerus, is one which must present itself occasionally to every surgeon in extensive practice, and for the satisfactory solution of which the records of the science do not as yet contain a sufficient number of well observed facts.

Thus there are numerous cases of reduction after a lapse of several weeks, or even months, without serious effects, Dupuytren succeeded in reducing them at 49, 51, 60, 82, 90, and even at 92 days. But he refused to attempt the reduction after two years, and advised the patient not to admit of such efforts. Sir Astley Cooper also reports cases of successful reduction at advanced periods, but limits the time at which they may be judiciously made to three months.

M. Sédillot reduced a dislocation under the spine of the scapula of one year's standing. Cases of reduction after two or three months had elapsed, are very numerous and have occurred in the practice of Physic, Dorsey, Gibson, &c., of our own country. After nearly six months, in that of Dr. McKenzie, of Baltimore, Mr. Kirby, of Dublin. It might be supposed that these authorities and cases were sufficient to determine the question. But on the other hand, we find the opinion of Boyer to be that "it is very rare that at the end of a month a dislocation, even of an orbicular articulation will be found capable of reduction." "Although we may have reduced some at the end of six weeks, two months, or even a longer time, we are far from thinking that these rare and happy cases can serve as a general rule." We find in confirmation of this opinion, that such efforts in the hands of the most eminent surgeons, have often, probably in a large majority of cases, been unsuccessful, and they have desisted from their efforts as soon as these had been carried as far as prudence would permit.

Where efforts were persevered in, after well directed force failed for a length of time to remove the bone from its situation, the results have been often injurious, and in some instances fatal. Thus, a case has come within my knowledge, of extensive laceration of the skin, without movement of the dislocated bone, several of extensive ecchymosis and inflammation, and the cases on record, of this kind, are numerous. Instances of rupture of the muscles, tendons, arteries or nerves, followed by loss of the functions of the member, or by death, are not wanting. In a case of eleven days standing, the bone was replaced, after two trials, by

Mr. Leudet, the axillary artery was ruptured and the patient died. In the second case reported by Flaubert, the axillary plexus of nerves was believed to have been injured, and in another, this plexus was found to have been torn from the spinal cord. A case in which the axillary artery and muscles were ruptured, is mentioned by Charles Bell, and one by Sir Astley Cooper, in his work on Dislocations. Another, in which the artery was in the same state, occurred in the practice of Delpech. Velpeau, in a clinic at La Charité, April 4, 1840, stated that a similar case had occurred at Paris, and if I am not misinformed, one has since occurred in that city, under the care of this distinguished surgeon himself. The two cases of Prof. Gibson, in both of which the artery was ruptured, in which death resulted, are familiar to the profession in this country. To all these cases, may be added another which occurred in this city in 1840. After violent and unsuccessful efforts to reduce a recent dislocation of the shoulder, inflammation and gangrene ensued; amputation at the scapulo-humeral, articulation was performed and the patient died during the operation. In this case there was reason to impute the death to want of skill on the part of the surgeon. It will thus be seen that eleven cases of death, or serious injury from attempts to reduce dislocations of the shoulder are easily found on record. It is probable there are several others which have not come within my knowledge.

Before concluding, it may not be useless to glance at some of the circumstances which should influence us in making attempts at reduction.

1. The extent of the injury. Ill effects are less liable to follow efforts at reduction of a simple dislocation, than one complicated with extensive injury of the surrounding parts. The difficulty and danger are much greater where inflammation has followed the accident.

2. The age and state of the patient. In old persons, and those of enfeebled constitutions, adhesions are not formed so easily as in the young and vigorous. In these latter too, the resistance of the muscles is more prompt and powerful.

3. The extent of the displacement. When the head of the bone is far removed from the socket, or if the limb is greatly shortened, the difficulty and danger are much greater than when the displacement is slight. It is probable that the state of the member in regard to mobility has an influence; when it can be freely rotated and extensively moved, the chances of success are more numerous than in the opposite conditions. Taking all these circumstances into consideration, an opinion may be formed with considerable certainty, of the result of an attempt at reduction. There are other circumstances, however, which can only be ascertained by a trial; and if on the application of a certain degree of force, the head of the bone does not move, if on making the movements of rotation, and pressing it in different directions, the adhesions do not yield, it is better to leave the case to the efforts of nature, for restoration of the usefulness of the member.

I am much indebted to Dr. B. P. Crossman, of Galena, Ill., for his assistance in the reduction of the above dislocation, without which, I might have had much more difficulty in effecting it.

